# Sporadic Outbreaks of Avian Infectious Bronchitis Viruses Highly Similar to the S95 Live Attenuated Vaccine Strain in Japan: A Comparative Study of Ten Field Isolates and S95

**DOI:** 10.3390/vaccines13111092

**Published:** 2025-10-24

**Authors:** Ryohei Nukui, Mari Takahashi, Atsushi Kato, Shiori Oguro, Erika Tanahashi, Takashi Ohmori, Nobuyuki Tsutsumi

**Affiliations:** 1 Nisseiken Co., Ltd., 9-2221-1 Shin-machi, Ome, Tokyo 198-0024, Japan; r.nukui@jp-nisseiken.co.jp (R.N.);; 2 Nippon Institute for Biological Science 9-2221-1 Shin-machi, Ome, Tokyo 198-0024, Japan

**Keywords:** avian, infectious bronchitis virus, IBV, live attenuated vaccine, vaccine marker

## Abstract

Background: As infectious bronchitis virus (IBV) strains similar to the IBV S95 live attenuated vaccine strain have been occasionally detected in poultry farms in Japan, we investigated the suspicion that outbreaks of the disease were related to the S95 vaccine. Methods: We isolated ten S95 vaccine-like strains, classified in the JP-I genotype of S1, the VIb (Y-4) genogroup of S2, and the GI-18 lineage, from IBV-affected chickens in Japan between 2020 and 2024. The whole-genome sequence and adaptation to embryonated chicken eggs were investigated. We developed a method for distinguishing the S95 vaccine strain from S95-like wild-type strains using specific primer sets having either the S95 vaccine or S95 parent-specific nucleotide at the 3′ termini of primers on the *ORF2* gene. Results: Nine of ten S95 vaccine-like strains lacked identical mutations to the *ORF1ab*, *ORF2*, and *ORF5a* genes that the S95 vaccine strain acquired during attenuation. The remaining S95-like strain, B3389, had identical mutations to the S95 vaccine strain in the *ORF1ab* and *ORF5a* genes. The B3389 strain, however, had strain-specific nucleotides that were not found in the S95 vaccine or S95 parent strains, and produced fewer embryonated egg-adapted phenotypes than the S95 vaccine strain. Conclusions: The ten S95-like strains appear not to have emerged from the S95 vaccine strain. Instead, sporadic outbreaks of S95 vaccine-like IBV strains in Japan were indicated. A method for distinguishing and excluding the S95-like wild-type strains as suspected revertants of the S95 vaccine may be utilized for comprehensive IBV surveillance to facilitate development of a vaccination strategy.

## 1. Introduction

Infectious bronchitis (IB), caused by the infectious bronchitis virus (IBV), is a highly contagious chicken disease characterized by respiratory disease and nephritis, which can lead to death from acute infection with a secondary infection [1]. IBV is transmitted via respiratory secretions and fecal droplets from infected chickens [1]. IBV primarily replicates in the upper respiratory tract and secondarily in the lower respiratory tract and non-respiratory epithelial cells of the alimentary tract, kidney, and gonads. IBV causes various symptoms depending on the type of infected tissue [2] and hampers egg production of laying hens and growth of broiler chickens, leading to economic losses in the poultry industry worldwide [3,4,5,6,7]. Chickens of all ages and breed types are susceptible to IBV; however, the severity of IB is thought to decrease with age [8].

The IBV is an enveloped, non-segmented positive-sense RNA virus belonging to the genus *Gammacoronavirus*, family *Coronavirinae*, order *Nidovirales* [9]. The IBV genome is 27–28 kb in length and contains 12 genes in the following order: 5′-ORF1a/1ab (Replicase, R)-2 (Spike, S)-3a-3b-3c-4a (Envelope, E)-4b (Membrane, M)-5a-5b-6a (Nucleocapsid, N)-6b-3′. ORF1ab is translated by a ribosomal frame-shift caused by a pseudoknot [10]. The main structural proteins of IBV are S, E, M, and N. Other proteins, including R, are non-structural proteins. ORF 1a/1 ab protein is further cleaved into 15 nonstructural proteins (NSP2-16) like other coronaviruses but lacks the NSP1 protein [11,12,13,14,15].

Compared to other coronaviruses, IBV lacks the NSP1 protein. The S protein, a highly glycosylated class I fusion protein, is located on the surface of the virion and plays a role in viral attachment to host cells and fusion with the host cell membrane or the vesicle membrane in the host cell cytoplasm [16]. The S protein is post-translationally cleaved at a basic amino acid-rich cleavage site into S1 and S2 protein subunits. The S1 subunit represents major antigenic epitopes for virus-neutralizing antibodies; therefore, mutations that have occurred in the S1 subunit (discussed below) have promulgated the emergence of variant viruses that sometimes exhibit distinct cell or tissue tropisms [17].

Similar to other RNA viruses, IBV is characterized by a high mutation rate, caused by the error-prone RNA replicase, and easy recombination enabled by the unique transcription and replication machinery common to coronaviruses. Both of these processes result in changes in antigenic properties, tissue tropism, pathogenicity, and, ultimately, the course of the disease [6,18,19]. Recombination occurs when two different IBV strains infect the same cell simultaneously [6,20]. A variety of IBV strains have emerged due to the intrinsic high mutation rate. IBV has been classified using the *S* gene sequence of the RT-PCR product, as most nucleotide variations are concentrated in the *S* gene, probably due to immunological selective pressure.

Several genotyping approaches have been used on IBVs within a limited geographical area, including China [6,21,22], Korea [5,23], and Japan [24,25,26,27,28,29,30]. In Japan, genotypes of IBVs had been classified either using a portion of the *S2* gene part into I to V, VIa, VIb (alternatively referred to as the Y-4, after the representative IBV Y-4 strain), and VII to VIII genogroups [27,31,32] or using a portion of the *S1* gene part, into Massachusetts, Gray, Connecticut, JP-I, JP-II, JP-III, JP-IV, and 4/91 genotypes [24,25,26,27,28,29]. Based on the entire *S1* gene sequences, IBV is now globally classified into six genotypes (GI to GIV), comprising 32 distinct lineages (GI-1 to GI-27, GII-1 to GII-2, GIV-1, GV-1, and GVI-1) [33].

It is crucial to maintain farm sanitation and hygiene and to prevent the introduction of diseases, including IB. Thus, vaccination is recommended for controlling IB in poultry houses. At least seventeen inactivated and live attenuated vaccines have been licensed as representatives of each S1-based genotype designated in Japan and used since 1969. Among the vaccines, the live attenuated IBV vaccine Nobilis 4-91 (MSD Animal Health Japan, Tokyo, Japan), associated with the 4/91 genotype of S1 and the VIII genogroup of S2, was actively used to control the newly emerged 4/91 genotype IBVs in Japan. Isolation of the highly similar wild-type IBV strain (JP/Wakayama-2/2004) from Nobilis 4-91 vaccinated chicken suggested the possibility of an emergence of vaccine-derived virulent IBVs [34]. However, the possibility of emergence was repudiated by the absence of the vaccine-specific restriction enzyme *Bgl* II site in the JP/Wakayama-2/2004 strain. Recently, IBVs sharing a similar S sequence with the S95 live attenuated vaccine strain, which was approved in 2013 as S95-IB (Nisseiken Co. Ltd., Ome, Japan) and is now commonly used in Japan, have been detected sporadically in IB-affected chickens in Japan.

In this study, we identified ten S95 vaccine-like IBV strains belonging to JP-I of S1 genotype, VIb (Y-4) of S2 genogroup, and GI-18 lineage collected from IB-affected chickens in poultry houses either after administration of the S95 vaccine or independently of S95 vaccination. We examined the whole-genome sequence of the ten S95-like IBV strains, as well as the S95-live attenuated vaccine strain and the S95-virulent parent strain. Among the ten strains, we selected the most similar strain to the S95 vaccine strain. We compared its phenotype, such as chicken embryo mortality, growth kinetics, and replication capacity in the embryonated chicken eggs, with those of the S95-live attenuated vaccine strain and the S95-virulent parent strain. Our findings may be crucial for evaluating the potential of the attenuated vaccine virus to revert to its wild-type virulence. Further, we propose a method for differentiating the S95 vaccine strain from S95-like wild-type strains.

## 2. Materials and Methods

### 2.1. IBV S95 Isolate and Its Attenuation History

The IBV S95 strain was initially isolated from the kidney of a chicken that showed nephritis in a laying hen house in Saitama Prefecture, Japan, in 1995. The S95 virulent progenitor virus was then passaged more than 40 times through embryonated chicken eggs via chorioallantoic cavity inoculation. A portion of the early passaged sample was stocked as the S95 parent strain (S95-E4) for reference. These successive passages were followed by several passages through primary chicken kidney (CK) cell cultures. The virus was plaque-purified on a CK cell culture and named the S95-P7 strain. It was amplified in embryonated chicken eggs several times to produce the seed virus for the live attenuated vaccine (S95-IB, Nisseiken Co., Ltd.), which had an avirulent character in chickens but grew well in eggs. S95-IB was approved for manufacturing and sales by the relevant authority in 2013.

### 2.2. IBV S95-Like Strain Isolation and Propagation

Ten field samples (B3024, B3273, B3362, B3364, B3389, B3510, B3539, B3616, B3639, and B3691) were collected from the kidneys of dead chickens from different poultry houses from 2020 to 2024. They were tested by RT-PCR using a 400 bp-length fragment of the IBV S2 following subsection and found to have an identical amplified DNA sequence to that of the S95 vaccine strain. Thus, we referred to these strains as S95 vaccine-like strains belonging to the VIb (Y-4) genogroup of S2. Six of the ten samples, B3273, B3362, B3389, B3539, B3616, and B3691, had an S95-IB vaccination history, but the remaining four did not (Table 1). Mean and median ages at sampling (due to death) were 38.9 and 35.0 days, respectively. To obtain the IBV from the kidney samples, 100 µL of 10% homogenate was inoculated into four to six 11-day-old embryonated SPF chicken eggs through the chorioallantoic cavity route. Eggs were incubated at 37.0 °C for two days and candled daily to determine if the embryos had survived. The allantoic fluid of the eggs that survived was harvested. IBV growth in the allantoic fluid of the eggs was confirmed by RT-PCR after the fluids were pooled. This egg passage was repeated three times. The third passage of each sample was pooled, and the viral titer was expressed as the 50% egg infectious dose (EID_50_) determined by inoculating embryonated chicken eggs with 10-fold serial dilutions of the samples. Embryonated eggs used in this study were obtained from specific-pathogen-free (SPF) white leghorn chickens (Line-M, Nisseiken Co., Ltd., Hokuto, Japan).

### 2.3. Conventional RT-PCR for the S2 Region of IBV

Kidney samples were temporarily stored at −80 °C until subsequent isolation of IBV RNA, when they were weighed and then mixed with nine volumes (converting 1 g to 1 mL) of phosphate-buffered saline containing penicillin and streptomycin (1000 μg/mL each). Samples were homogenized in a tissue blender and then centrifuged (9100× *g* for 10 min). The supernatants were designated as 10% (*w*/*v*) tissue homogenates. When the allantoic fluids of the eggs were used, the fluids were applied to the RNA extraction procedure after low-speed centrifugation (800× *g* for 10 min). Viral RNA was extracted and purified using the RNA tissue Kit SII (FUJIFILM Wako Pure Chemical Co., Tokyo, Japan) and QuickGene-Mini80 (FUJIFILM Wako Pure Chemical Co.) according to the manufacturer’s instructions. DNA fragments were amplified using the one-step PrimeScript RT-PCR kit (Takara Bio Inc., Kusatsu, Japan). To amplify the 400 bp-length DNA fragment in the S2, the original forward primer [31,32] and modified reverse primer for S2 (5′-ARYAARCCATTATAYTCWCGRGCAC-3′) [30] were used. The primer set and RNA were pre-incubated at 94 °C for 2 min and then chilled on ice. The reaction mixture was then added, and the reverse transcription was performed at 45 °C for 30 min, followed by heat inactivation at 94 °C for 2 min. The DNA fragments were then amplified for 40 cycles at three-step temperatures comprising 94 °C for 15 s for denaturation, 50 °C for 30 s for annealing, and 72 °C for 30 s for extension.

### 2.4. Quantitative Real-Time RT-PCR for the 5′ Untranslated Region of IBV

The conserved 5′ untranslated region (UTR) of the IBV was used to amplify a 143 bp-length DNA fragment using the forward primer GU391 (5′-GCTTTTGAGCCTAGCGTT-3′), the reverse primer GL533 (5′-GCCATGTTGTCACTGTCTATTG-3′), and the Taqman probe IBV G (5′-FAM-CACCACCAGAACCTGTCACCTC-BHQ1-3′), which are, respectively, located at nucleotide positions 392–409, 534–513, and 495–474 of the IBV C-78E128 strain genome (GenBank accession no. LC663496) as designed by Callison et al. [35]. The reaction was carried out using a StepOne Plus real-time PCR system (Applied Biosystems, Thermo Fisher Scientific K.K., Tokyo, Japan) at 50 °C for 5 min, followed by 95 °C for 20 s, and then 40 cycles at 95 °C for 3 s and 60 °C for 30 sec. As a quantitative standard RNA, runoff RNA transcript corresponding to nucleotide positions 392–474 was synthesized using T7 RNA polymerase (MEGAshortscript T7 Transcription Kit, Thermo Fisher Scientific K.K.) and the DNA template. The DNA template for the in vitro RNA synthesis was prepared by amplifying the DNA fragment using the forward primer 5′-taatacgactcactatagggaga-GU391-3′ (the underlined part indicates the T7 promoter sequence), the reverse primer GL533, and the IBV C-78 complete genome cDNA [36], followed by purification with NucleoSpin Gel and PCR Clean-Up (Takara Bio Inc.). Following DNase I (included in the T7 Transcription Kit) treatment to remove the template DNA, the synthesized RNA was purified using the MEGAclear Transcription Clean-Up Kit (Thermo Fisher Scientific K.K.). The quantity of RNA was measured in µg/µL at an absorbance of OD260 using Nano Drop 2000c (Thermo Fisher Scientific K.K.), and was converted to the copy number/µL by its nucleotide base numbers.

### 2.5. Sequencing of IBV S95-Like Strains

After purifying DNA fragments using a Nucleospin Gel and PCR Clean-Up kit (Macherey-Nagel Takara, Tokyo, Japan), DNA sequencing was performed by a sequence service (Fasmac Co., Ltd., Atsugi, Japan). Whole-genome sequences of each IBV were initiated using viral genomic RNAs extracted by a High Pure Viral RNA Kit (Roche Diagnostics K.K., Tokyo, Japan) or a NucleoSpin RNA Clean-Up (Takara Bio Inc.). The virulent S95-E4 parent strain was sequenced as described previously [36]. The live attenuated S95 vaccine strain and ten S95-like field strains were sequenced by applying random, non-targeted next-generation sequencing service with the Illumina MiSeq sequencer (Illumina Japan Co., Ltd., Tokyo, Japan).

### 2.6. Capacity of IBV S95-Like Strains to Kill Chick Embryos

The capacity for the IBV S95-E4 parent strain, S95 vaccine strain, or B3389 strain to kill chick embryos was measured by inoculating each strain into 9-day-old embryonated SPF chicken eggs. Forty eggs were inoculated either with 10 EID_50_/0.1 mL of each strain or PBS (as a control) through the chorioallantoic cavity route and incubated for 7 days at 37 °C. Ten eggs inoculated with either the strain or PBS were candled every day to see if the embryo within each egg was alive or dead, and the data were compared using a Student’s *t*-test (*p*-values of <0.05 were considered to indicate significant differences). Two eggs were incubated for 7 days without any inoculation as a non-treatment control. After 7 days of incubation, live embryos were taken from the eggs and compared macroscopically. We repeated this study to confirm the results.

### 2.7. Growth Kinetics and Replication Capacity of IBV S95-Like Strains in Embryonated Chicken Eggs

Twenty-five 9-day-old embryonated SPF chicken eggs were inoculated either with 10 EID_50_/0.1 mL of IBV S95-E4 parent strain, S95 vaccine strain, or B3389 strain through the chorioallantoic cavity route and were incubated at 37 °C. Five eggs inoculated with each strain were taken from the incubator at 6 h, 18 h, 24 h, 30 h, and 48 h post-inoculation, and the allantoic fluids of the eggs were used for extracting the viral RNA. The viral genome copy number at each time point for each inoculation group was determined using real-time RT-PCR. Data were analyzed using a two-tailed Student’s *t*-test and were expressed as the mean ± standard deviation (SD) of independent experiments (*p*-values of <0.01 were considered significantly different). This study was repeated to confirm the results.

### 2.8. Sequence Alignment and Phylogenetic Tree Analysis of IBV S95-Like Strains

IBV sequences were compared to sequences of 87 relevant IBV strains collected from GenBank. Sequence alignment and a phylogenetic tree analysis were performed using the Muscle and maximum likelihood methods, respectively, with Genetyx-MAC (version 22, Nihon Server, Tokyo, Japan). The number of nucleotide substitutions was inferred using the Tamura–Nei model, with the Neighbor-Join and BioNJ algorithms applied and 1000 bootstrap replications performed. The tree was visualized and edited using the FigTree software (version 1.4.4).

### 2.9. PCR-Based Differentiation of the S95 Vaccine Strain and S95-Like Virulent Field IBVs

For differentiation of the S95 vaccine strain and the S95-like IBV strains, two primer pairs were prepared. A forward primer, IBV-S1WF, 5′-CGGCCCTCAATTTTGCCc-3′ (a lowercase letter shows the different nucleotides between differentiation primers) and a reverse primer, IBV-S1WR, 5′-CACGTTGCTTTGCCCGAa-3′ were used for S95-like field IBVs with a wild-type phenotype (W primer set). The forward primer, IBV-S1AF, 5′-CGGCCCTCAATTTTGCCt-3′ and the reverse primer, IBV-S1AR, 5′-CACGTTGCTTTGCCCGAc-3′, were used for the S95 vaccine strain with an attenuated phenotype (A primer set). Both primer pairs were designed to amplify a 777 bp-length DNA fragment located at nucleotide positions 351–1127 of the IBV S gene. The reaction was carried out using PrimeScript One Step RT-PCR Kit (ver. 2, Takara Bio Inc.) and a ProFlex PCR system (Applied Biosystems, Thermo Fisher Scientific K.K.) at 45 °C for 30 min for reverse transcription, followed by 94 °C for 2 min for denaturation, and then 40 cycles of three step PCR composed with 94 °C for 15 s of denaturation, 69 °C for 30 s of annealing, 72 °C for 30 s of extension, with subsequent further extension at 72 °C for 5 min. PCR products were electrophoresed on a 1.2% (*w*/*v*) agarose gel using TBE buffer (89 mM Tris-HCl, pH 8.3, 2 mM EDTA-2NA, and 89 mM Boric acid).

## 3. Results

### 3.1. Live Attenuated S95 Vaccine and S95-Like Strains in the Field

**Figure 1:**
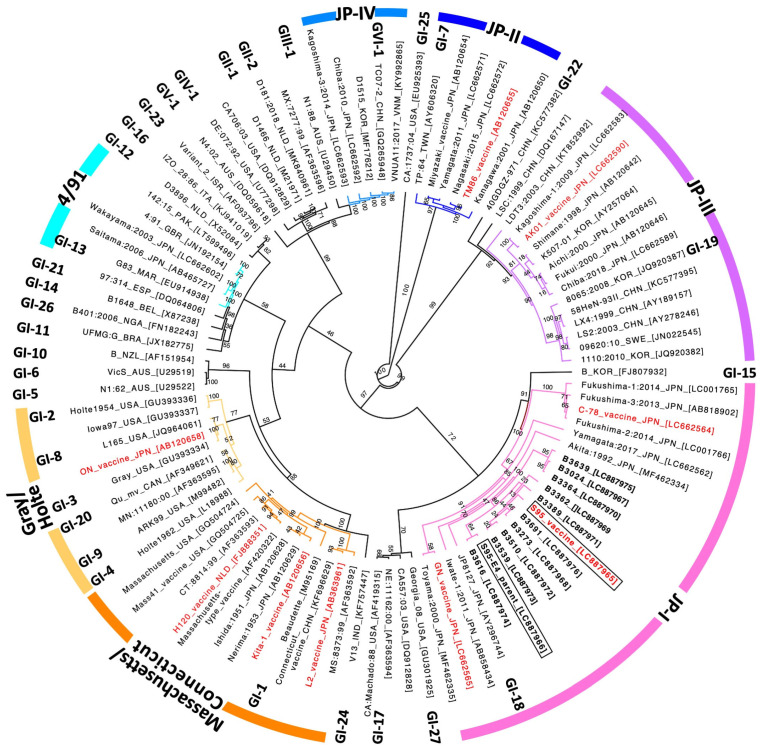
Phylogenetic analysis of the S gene sequence of IBVs. The phylogenetic tree of the ORF2 (S) gene was created with 87 representative IBV strains retrieved from the DNA databank. Ten S95-like strains, B3024, B3273, B3362, B3364, B3389, B3510, B3539, B3616, B3639, and B3691 are indicated in bold type. The S95-E4 parent strain and the S95 vaccine strain are indicated in boxes. Live attenuated IB vaccines used in Japan are indicated in red. Thirty-two distinct lineages (GI-1 to GI-27, GII-1 to GII-2, GIV-1, GV-1, and GVI-1) are indicated at their representative strain [33]. The IBV strains, classified into one of seven genotypes (Massachusetts, Gray, Connecticut, JP-I, JP-II, JP-III, JP-IV, and 4/91 [24]), are shown as differently colored branches of the phylogenetic tree. Bootstrap values calculated with 1000 replicates are shown in the branch of the phylogenetic tree.

IBV can change easily during transmission, a characteristic of coronaviruses, and so, to date, numerous IBV vaccines against newly emerged strains have been developed. The live attenuated S95-IB vaccine was licensed in 2013 in Japan and is used to control avian infectious bronchitis, particularly when accompanied by nephritis. Because S95 vaccine-like sequences were recently identified from IBV-affected chicken kidney samples, the possibility of reversion to virulence of a live attenuated S95 strain from the S95-IB vaccine has been discussed. To address the question, we collected kidneys from chickens, which had probably died as a result of IBV infection, from poultry houses from around the country (except for the northern Japan region) between 2020 and 2024. We then identified ten samples (B3024, B3273, B3362, B3364, B3389, B3510, B3539, B3616, B3639, and B3691) that had an identical sequence to the IBV S95 vaccine strain in the S2 amplified region of the *S* gene (Table 1). We then isolated the S95-like IBVs from the ten kidney samples. Whole-genome sequences of the ten S95-like strains [Gene accession numbers LC887967 (B3024), LC887968 (B3273), LC887969 (B3362), LC887970 (B3364), LC887971 (B3389), LC887972 (B3510), LC887973 (B3539), LC887974 (B3616), LC887975 (B3639), and LC887976 (B3691)] and the avirulent S95 vaccine strain [Gene accession number LC887965] were determined by using a random, non-targeted next-generation sequencing service (Fasmac Co., Ltd., Illumina). The virulent S95-E4 parent strain [Gene accession number LC887966] was sequenced using a direct sequencing method. The phylogenetic tree analysis based on the *S* gene with eighty-seven representative IBV strains retrieved from the DNA databank showed that the ten S95-like strains, the S95 vaccine, and the S95-E4 parent strains are classified into the JP-1 genotype, which is internationally categorized into the GI-18 lineage (Figure 1). Further, the ten S95-like strains were closely clustered with the S95 vaccine and S95-E4 parent strains in the phylogenetic tree (shown in bold type in Figure 1).

### 3.2. Comparison of S95-Like Strains with the S95 Vaccine Strain at the Gene Level

To examine the details of the genome sequence of S95-like strains, we compared the homology of eleven genes (excluding the *ORF1a* gene, which is included in the *ORF1ab* gene) of each strain using both nucleic acid and deduced amino acid sequences. Unfortunately, the mutations that occurred on the 5′ UTR and 3′ UTR were not used, because the reliability of the whole-genome sequences at both termini regions was not high. The S95 vaccine strain exhibited 100% identical nucleic acid and amino acid sequences to the virulent parent strain in 8 of the 11 genes when serving the sequence of the S95-E4 parent strain as the 100% reference (Table 2, top). Thus, only the *ORF1ab*, *ORF2 (S)*, and *ORF5a* genes of the S95 vaccine strain differ from the parent strain, while other genes are identical.

Among the ten S95-like strains, B3024, B3364, and B3539 were considered not to have emerged from the S95 vaccine strain because nucleotide mutations were found in all eleven genes. This suggests that they could not have evolved from the S95 vaccine strain because the time frame was too short for these differences to develop, and thus, these three strains were excluded from further analysis. When serving the sequence of the S95 vaccine strain as the 100% reference, the remaining six S95-like strains, except for the B3639 strain, have at least three genes identical to those of the S95 vaccine strain, including the *ORF3a*, *ORF3b*, and *ORF3c (E)* gene sequences (Table 2, bottom). The B3639 strain has an *ORF3a* gene that differs from that of the S95 vaccine strain; however, it possesses identical genes to those of the S95 strain in *ORF3b*, *ORF3c (E)*, *ORF4b, ORF5a, ORF5b*, *ORF6a (N)*, and *ORF6b*. Three S95-like strains, B3389, B3273, and B3510, were more similar to the S95 vaccine strain than others because, in addition to having the identical *ORF3a*, *ORF3b*, and *ORF3c (E)* genes, the deduced amino acid sequences of the *ORF4a(M)*, *ORF4b*, and *ORF5b* genes were also identical to the S95 vaccine strain.

### 3.3. Amino Acid Changes in ORF1ab (R), ORF2 (S), and ORF5a Genes Acquired by the S95 Vaccine Strain During Attenuation and Amino Acids in the Same Positions of S95-Like Strains

Five amino acid differences between the S95-E4 parent strain and the S95 vaccine strain were found in the ORF1ab RNA replicase protein, composed of 6633 amino acids: 3928th serine (T^11783^CT, as indicated by both codon and the position in the gene of the changed nucleotide) to phenylalanine (T^11783^TT), 3939th aspartic acid (^11815^GAT) to tyrosine (^11815^TAT), 4346th histidine (CA^13037^T) to glutamine (CA^13037^G), 4851st serine (A^14551^GT) to asparagine (A^14551^AT), and 5382nd threonine (A^16144^CA) to isoleucine (A^16144^TA) (Table 3). Two amino acid changes, 123rd proline (C^368^CA) to leucine (C^368^TA) and 370th phenylalanine (TT^1110^T) to leucine (TT^1110^G), and nine amino acid truncations in the carboxyl termini, with 1161st glutamic acid (^3482^GAA) changed to the *ochre* stop codon (^3482^TAA), were found in the ORF2 S protein, composed of 1169 amino acids. One amino acid change was identified in the ORF5a non-structural protein, which is composed of 65 amino acids, specifically a change of the 11th valine (G^32^TT) to alanine (G^32^CT). Some or all of these changes were considered to be related to the determinants of viral virulence.

To examine the differences between S95 vaccine strains and the seven S95-like strains, we focused on amino acid changes in the *ORF1ab (R)*, *ORF2 (S)*, and *ORF5a* genes, which were acquired by the S95 vaccine strain during attenuation from the S95-E4 parent strain in egg passages (Table 3). Five amino acids found in the *ORF1ab (R)* gene of the S95-E4 parent strain were fully conserved in the *ORF1ab (R)* gene of the B3273, B3510, and B3362 strains; four of the five amino acids were conserved in the B3639 and B3616 strains, and three amino acids were conserved in the B3691 strain. The changed amino acid (glycine, Gly) found in the *ORF1ab (R)* gene of the B3616 and B3691 strains differed from the amino acid (tyrosine, Tyr) found in the same gene of the S95 vaccine strain. Three amino acids found in the *ORF2 (S)* gene of the S95-E4 parent strain were also conserved in the *ORF2 (S)* gene of the B3389, B3510, B3362, B3616, B3639, and B3691 strains. One amino acid change found in the *ORF5a* gene of the S95-E4 parent strain was also present in the *ORF5* genes of the B3510, B3362, B3616, and B3691 strains. These results indicated that six of the seven S95-like strains, B3273, B3510, B3639, B3362, B3616, and B3691, maintained amino acids of the S95-E4 parent strain rather than those of the S95 vaccine strain. We considered that the six strains (which excludes the B3389 strain) were not revertants of the S95 vaccine strain but were wild-type IBV strains in the field, because the chances of the rare event of amino acids located in different portions of *ORF1ab (R)*, *ORF2 (S)*, and *ORF5a* genes reverting to those of the S95-E4 parent strain in the time frame is scarce. However, the B3389 strain has the closest relationship to the S95 vaccine strain, with five amino acid changes in the *ORF1ab (R)* gene of the S95 vaccine strain and one amino acid change in the ORF5a entirely conserved in the B3389 strain (Table 3). The close relationship between the B3389 strain and the S95 vaccine strain was further indicated by their location in the *S* gene-based phylogenetic tree (Figure 1 and Table 2).

### 3.4. Egg Embryo Mortality Associated with the B3389 Strain

**Figure 2:**
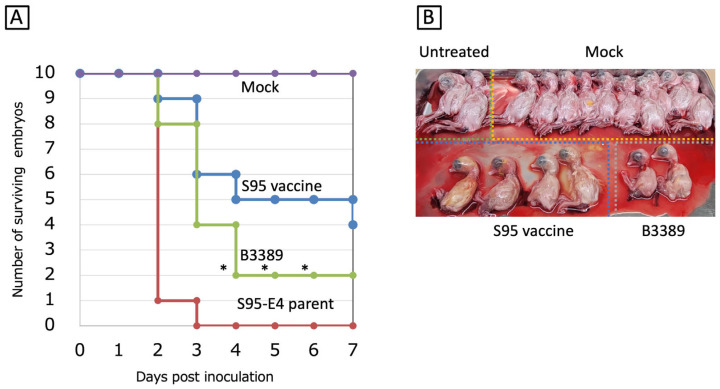
Egg embryo mortality associated with the B3389 strain. (**A**) Three strains, S95 vaccine-like B3389, S95 vaccine, and S95-E4 parent, were inoculated into the allantoic cavities of ten SPF chicken eggs, and incubated for 7 days. Surviving egg embryos, judged by candling, were counted. Significant unpaired *t*-test (*n* = 10) results (*p* < 0.05) comparing mortality in the S95 vaccine strain and the B3389 strain inoculated eggs are indicated by *. (**B**) Macroscopic embryo lesions (stunting or embryo dwarfing) were observed at the end of the incubation periods. Ten PBS-infected eggs and two non-treated eggs were used as controls and baseline comparisons.

Among the ten S95-like strains, the B3389 strain was the most similar to the S95 vaccine strain. We therefore investigated the egg embryo mortality rate caused by the S95-like B3389 strain to determine if it possessed the embryonated chicken egg-adapted property that resides in the S95 vaccine strain. Nine of ten embryos (90%) were killed at 2 days post-inoculation, and ten embryos (100%) were killed at 3 days post-inoculation by the S95-E4 parent strain (Figure 2A). The S95 vaccine strain killed one, three, one, and one embryos at 2, 3, 4, and 7 days post-inoculation, and thus six out of ten embryos (60%) were killed during the experimental period (Figure 2A). The S95 vaccine strain showed less mortality in eggs than the S95-E4 parent strain. Under these experimental conditions, the S95-like B3389 strain killed two, four, and two embryos at 2, 3, and 4 days post-inoculation. Thus, eight out of ten embryos (80%) were killed during the experimental period (Figure 2A). Significant differences (*p* < 0.05) between the mortality of the S95 vaccine strain and the B3389 strains were observed at 4, 5, and 6 days post-inoculation. Four embryos inoculated with the S95 vaccine strain survived and were nearly the same size as those of the control inoculates. However, the two embryos inoculated with the S95-like B3389 strain that survived appeared smaller than the others (Figure 2B). Thus, although mortality associated with the B3389 strain (80%) was lower than that of the S95-E4 parent strain (100%), the strain retained the virulent phenotype for the egg embryo.

### 3.5. Replication Capacity of the B3389 Strain in Embryonated Chicken Eggs

**Figure 3:**
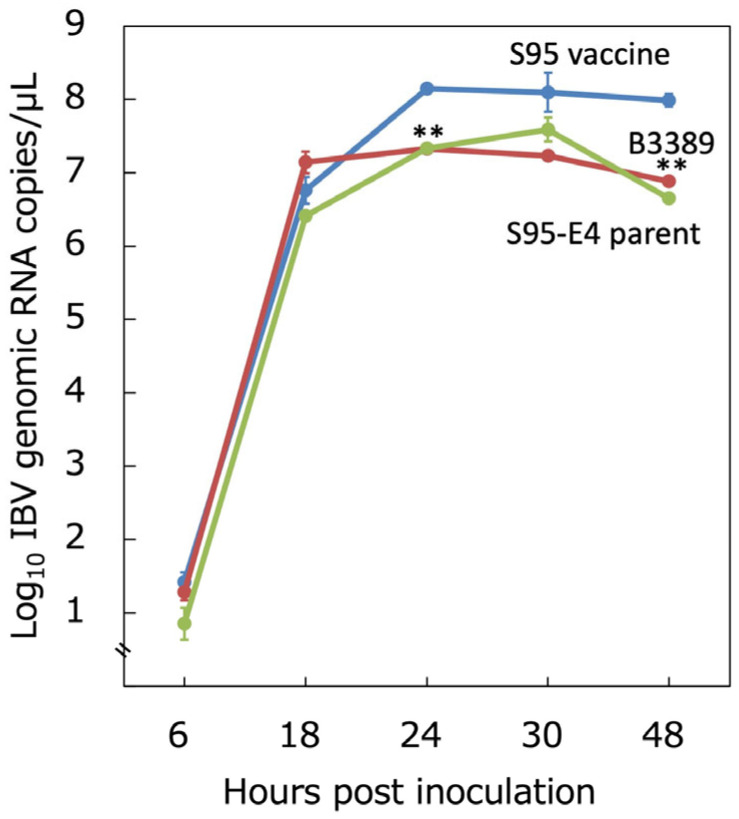
Replication capacity of the B3389 strain in embryonated chicken egg. Three strains, S95 vaccine-like B3389, S95 vaccine, and S95-E4 parent, were inoculated into the allantoic cavities of ten SPF chicken eggs. Allantoic fluid containing the virus was evaluated for the number of IBV genomic RNA copies using real-time RT-qPCR at 6, 18, 24, 30, and 48 h post-inoculation. Error bars indicate the mean ± SD. Significant unpaired *t*-test (*n* = 3) results (*p* < 0.01) between the development of the S95 vaccine strain and that of the B3389 strain are indicated by **.

We further examined the replication capacity of the S95-like B3389 strain in embryonated chicken eggs to determine if it had adapted to develop within them. The virus titer in the allantoic fluid was evaluated using a real-time RT-PCR as the genome copies/µL. The S95 vaccine, S95-E4 parent, and B3389 strains replicated to 10^6.76±0.18^, 10^7.14±0.15,^ and 10^6.41±0.02^ copies/µL, almost equally, by 18 h post-inoculation (Figure 3). The S95 vaccine replicated further and reached 10^8.15±0.03^ copies/µL, while the S95-E4 parent and B3389 strains remained at 10^7.33±0.02^ and 10^7.33±0.02^ copies/µL at 24 h post-inoculation. The S95 vaccine reached 10^8.10±0.27^ and 10^7.99±0.09^ copies/µL at 30 h and 48 h post-inoculation, respectively. However, the S95-E4 parent and B3389 strains were at 10^7.24±0.04^ and 10^7.59±0.16^ copies/µL at 30 h post-inoculation, and 10^6.88±0.06^ and 10^6.65±0.00^ copies/µL at 48 h post-inoculation. The development of the S95 vaccine strain and the B3389 strain was significantly different (*p* < 0.01) at 24 and 48 h post-inoculation. Development associated with the S95-E4 parent and the B3389 strains did not differ significantly. Thus, the S95-like B3389 strain exhibited a replication capacity lower than that of the S95 vaccine strain, but similar to its parent strain, S95 (Figure 3). In other words, the S95-like B3389 strain was less adapted in embryonated chicken eggs than the S95 vaccine strain.

### 3.6. Differentiation of the S95 Vaccine Strain from the S95-Like Field IBV Strains

**Figure 4:**
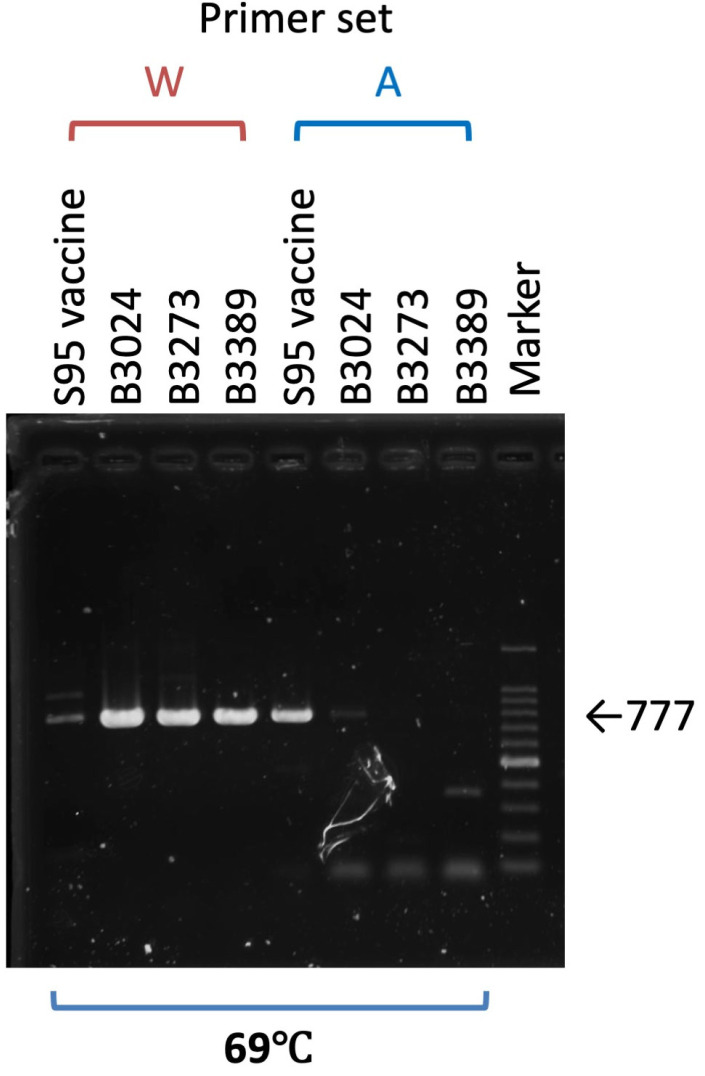
Differentiation of the S95 vaccine strain from the S95-like wild-type IBV strains. Viral RNAs extracted from three S95-like wild-type strains, B3024, B3273, and B3389, and the S95 vaccine strain were either amplified with the specific primer set W and have the S95-E4 parent strain-specific nucleotide at the 3′ terminus or with the primer set A and have the S95 vaccine strain-specific nucleotide at the 3′ terminus to give the 777 bp-length DNA band. An agarose gel electrophoresis image of amplified DNA is shown. The marker indicates the 100 bp ladder DNA. A thick band of the marker lane in the agarose gel shows the 500 bp-length DNA.

The possibility of a virulent virus emerging from the live attenuated vaccine at the developmental stage was refuted by a virulence revision test. However, it is crucial to develop a method for differentiating wild-type strains from live attenuated vaccines. As indicated above, we identified three nucleotide changes, ^368^C to T, ^1110^T to G, and ^3482^G to T, that accompany the amino acid change in the *ORF2 (S)* gene of the S95 vaccine strain (Table 3). Since two of the three changes are located across a suitable distance, we designed two primer sets that have a nucleotide mutation at the 3′ terminus of the primer facing each other. One primer set has an S95-E4 parent strain-specific nucleotide at the 3′ terminus (^368^C and ^1110^T), and the other set has a S95 vaccine strain-specific nucleotide at the 3′ terminus (^368^T and ^1110^G). Both were designed to produce a 777 bp-length DNA fragment following PCR amplification. After examining several temperature conditions for annealing, we found that 69 °C was the most suitable temperature for differentiating the S95 vaccine strain from the S95-like field IBV strain. Under this annealing temperature condition, the S95-like B3024, B3273, and B3389 strains were amplified by the W primer set (shown as W in Figure 4). They featured the S95-E4 parent strain-specific nucleotide at the 3′ terminus, and showed clear 777 bp-length DNA bands, while the S95 vaccine strain produced only a faint band. In contrast, the S95 vaccine strain had a S95 vaccine strain-specific nucleotide at the 3′ terminus and produced a clear band with the A primer set (shown as A in Figure 4) while the S95-like B3024, B3273, and B3389 strains produced only faint and short bands. These results indicate that the S95-like field IBV strains with the wild-type phenotype were detected by the W primer set, and that the S95 vaccine strain with the attenuated phenotype was detected by the A primer set.

## 4. Discussion

Vaccination is the most effective way of controlling IB in poultry houses. However, the emergence of IBV strains with antigenic variations makes IBV control difficult. To date, many IBV serotypes have been identified using the virus neutralization (VN) test performed in laboratories [26,29,36,37]. Generally, the VN test is complex and time-consuming. More importantly, as there are no established common VN standards, comparison between different laboratories’ VN tests is impossible. In contrast, the virus genotyping method using PCR does not require standards and takes less time, so IBV classification based on the *S* gene is widely accepted and used worldwide [33]. In Japan, research has indicated that concordance between S1 genotyping and serotype classification was approximately 65% [24]. The *S* gene-based classification can match a spreading IBV strain with the vaccine strain. Thus, genotyping of the *S* gene is helpful for accurate diagnosis, which leads to suitable vaccination planning and scheduling. Nevertheless, the administered vaccine does not always effectively control the prevalent wild-type IBV strain, even though a suitable vaccine recommended from the genotyping is used. This is partly explained by the timing of vaccination (if given too late to gain sufficient immunity) or by sanitation of the poultry house (if sanitation is insufficient to maintain healthy conditions) [5,38].

In this study, we collected ten S95-like strains from all over the country (excluding northern Japan). Among these, six strains (B3273, B3362, B3389, B3539, B3616, and B3691) were collected from IB-affected chickens from poultry houses in which the S95-IB vaccines are administered (Table 1). All strains fall within the same S1 JP-I genotype and S2 VIb genogroup as the S95 vaccine strain. Given that the genotype and genogroup of the wild-type IBVs obtained in poultry houses are the same as those of the IBV vaccine administered, the vaccine should be effective against the IB. Nonetheless, S95-like strains were isolated from the IB-affected chickens. This was sufficient evidence to suspect the occurrence of a virulent revertant of the live attenuated S95 vaccine strain. Vaccines are generally considered safe due to thorough evaluation and guarantees during the approval process by the relevant authority. In particular, the emergence of a virulent virus from live attenuated vaccines is of significant concern and is usually addressed before vaccine approval. While the possibility of a virulent strain emerging from live attenuated vaccine is low, it cannot be readily claimed to be zero. In fact, there are several reports of a virulent virus emerging from live attenuated vaccines [34,39].

We concluded that of the ten S95 vaccine-like strains, nine were not derived from the S95 vaccine strain because they possess unique mutations that are neither shown in the S95 parent nor the vaccine strain (Table 2), and do not possess the nucleotide mutations that the S95 vaccine strain acquired during the serial passages of attenuation (Table 3). Notably, the tenth strain, B3389, shares some mutations with the S95 vaccine strain in the *ORF1ab (R)* gene and the *ORF5a* gene. Since the B3389 strain had the most significant potential to be a virulent revertant, we used replicated experiments to confirm once more its poorer developmental ability in the embryonated chicken eggs (Supplemental Figure S1) and higher chick embryo mortality given its presence (Supplemental Figure S2). The discussed results (Figure 2 and Figure 3) and those in the replicated experiments (Supplemental Figures S1 and S2) collectively indicate that the B3389 strain lacks the chick embryo adaptation acquired by the S95 vaccine strain, along with its attenuation. However, the B3389 strain was unlikely to have lost full replicating capacity in eggs and reduced chick embryo mortality in exchange for generating virulence in chickens in the available (short) period. For this reason and because the B3389 strain has strain-specific mutations in the *ORF1ab(R)* and *ORF2(S)* genes in addition to mutations in common with S95 vaccine strain (Table 2 bottom), we concluded that the B3389 strain did not emerge from the S95 vaccine strain, but is a wild-type strain originating from a common ancestor, the S95-E4 parent strain. The virulence of the B3389 strain for chickens was not examined during this study due to practical constraints. Therefore, the isolated B3389 strain does not meet the criteria of Koch’s postulates; however, considering that it was obtained from the kidney of IB-affected chickens, it might have virulence in chickens. In conclusion, the ten investigated S95-like strains did not emerge from the S95 vaccine strain. Instead, our data suggest the sporadic occurrence in Japan of outbreaks of S95-like IBV strains that originate from a common ancestor with the S95-E4 parent strain and are classified in the JP-I genotype of S1, VIb (Y-4) genogroup of S2, GI-18 lineage.

Since we did not determine the sequences of the 5′ UTR and 3′ UTR regions of the IBV genome, we focused on differences in the *ORF1ab(R)*, *ORF2(S)*, and *ORF5a* genes between the S95 vaccine strain and the S95-E4 parent strain. One of the mutations occurred in the *ORF2(S)* gene and converted a glutamic acid at position 1161 to a stop codon, which truncated the C-terminal cytoplasmic tail of the S2 protein subunit by removing nine amino acids (Table 3). Truncation of the C-terminal cytoplasmic tail of the S2 protein during chicken embryo embryonation is thought to be involved in IBV attenuation [19,36,40]. Reverse genetic investigation indicates that this truncation resulted in the loss of the endoplasmic-reticulum-retention signal and indeed impaired the S protein from localizing to the endoplasmic-reticulum–Golgi intermediate compartment. Loss of localization significantly reduced the incorporation of the S protein into viral particles and reduced pathogenicity in chickens due to the reduction in viral invasion efficiency [19]. The *ORF5a* gene is not essential because a recombinant IBV that lacks expression of the ORF5a gene could be rescued and could develop in the chick primary kidney cells. Because it showed reduced pathogenicity in chickens [40,41,42], the *ORF5a* gene was, however, thought to be involved in the pathogenesis of IBV. Similar to other coronaviruses, the ORF1a/1ab protein of IBV is cleaved into NSP2 to NSP16 proteins. Among the five amino acid changes in Table 3, ^3928^Ser to Phe and ^3939^Asp to Tyr reside in NSP7, while ^4346^His to Gin resides in NSP9. On the other hand, the substitutions ^4851^Ser to Asn and ^5382^Thr to Ile occur in NSP10. Functions of several NSPs are reported [11,13,14,15,43], but the importance of amino acid changes is not yet evident. To understand the efficacy and genetic stability of the S95 vaccine strain, we sought to investigate the mutated *ORF1ab(R)*, *ORF2(S)*, and *ORF5a* genes, as well as the sequences of the 5′ UTR and 3′ UTR regions.

New IBV strains are known to emerge that are characterized by different antigenic properties and distinct tissue tropism based on the common property of coronaviruses. Emergence of new IBVs is partly accelerated by immunological selection induced by inactivated and live-attenuated vaccines administered for the control of IB. However, the use of live-attenuated IBV vaccines complicates identifying prevalent IBV strains by differentiating vaccine strains from vaccine-like wild-type IBV strains [44]. Therefore, it is beneficial to develop live-attenuated vaccine-specific markers. In this study, we identified an S95-specific genetic marker and demonstrated its ability to distinguish the S95 vaccine strain from S95-like field isolates using PCR. This marker should aid in assessing the prevalence of S95-like strains in poultry houses. As discussed, the *S* gene-based classification is being applied in Japan to identify whether the prevalent IBV strains match the vaccine strains to be used. For S1 classification, approximately 700 bp-DNA fragments, with diversity in lengths (671–692 bp) depending on the IBVs, are utilized [24,26,29]. This primer set covers nucleotide positions 25 to 717 of the *S* gene of the S95 vaccine strain. For S2 classification, a 400 bp-DNA fragment that covers nucleotide position 1829 to 2228 of the *S* gene [30,31,32] or a 490 bp-DNA fragment that covers nucleotide position 1829 to 2318 of the *S* gene of the S95 vaccine strain [24] is utilized. Three markers in the *S* gene of the S95 vaccine strain are ^368^C to T, ^1110^T to G, and ^3482^G to T mutations. Among these, the ^368^C to T mutation can be identified during the S1 classification covering nucleotide positions 25 to 717 of the *S* gene of the S95 vaccine strain. However, other markers cannot be identified during the S1 or S2 classification because they are outside the area. Therefore, a specific primer set that can differentiate the S95 vaccine strain from the S95-like field IBV strains is beneficial.

To evaluate possible revertants, it is essential to investigate both genetic and viral characteristics, such as pathogenicity. For instance, the S95 vaccine strain, the S95-E4 parent strain, and the S95-like B3389 strain had almost identical (over 99%) gene sequences but differed in virulence towards chick embryos. The PCR-based discrimination method that we demonstrated could be a valuable tool for evaluating the efficacy of the live attenuated S95 vaccine by distinguishing the emergence of S95-like wild-type IBVs. However, as our PCR method needs more verification using the field samples, we will continue to examine whether this PCR-based discrimination method can be used for assessing the prevalence of S95 vaccine-like IBVs in the field. Since none of the ten S95 vaccine-like strains used during this study were obtained from the northern part of Japan, it is necessary to continue monitoring to determine whether sporadic outbreaks of these strains occur throughout Japan. Continuous monitoring of live attenuated vaccine-like strains is also required for long-term and safe use of live vaccines in the field.

## Figures and Tables

**Table 1:** S95 vaccine-like IBV strains isolated in the poultry houses in Japan.

Field Sample	Year	Prefecture	Breed of Chicken	Disease State	Tissue	Days Old	IB Vaccination History
B3024	2020	Kagoshima	broiler	death	kidney	57	C-78, TM86, ON, Nerima
B3273	2021	Ibaraki	layer	death	kidney	28	GN, S95
B3362	2022	Gunma	layer	death	kidney	47	S95, AK01, H120, TM86
B3364	2022	Tokushima	broiler	death	kidney	35	Kita-1
B3389	2022	Okayama	layer	death	kidney	27	S95
B3510	2023	Aichi	layer	death	kidney	30	H120, H120
B3539	2023	Okayama	layer	death	kidney	36	C-78, S95, Ma5
B3616	2023	Okayama	layer	death	kidney	35	C-78, S95, H120, H120
B3639	2023	Aichi	layer	death	kidney	74	TM86, C-78, H120, AK01
B3691	2024	Kagawa	layer	death	kidney	19	S95, H120

Ten S95 vaccine-like IBV strains were isolated from the kidneys of dead chickens from different poultry houses from 2020 to 2024. The prefecture of Japan, breed of chicken, age (in days) at death, and IB vaccination history are indicated.

**Table 2:** Gene comparison of S95 vaccine-like IBV strains with S95 parent and vaccine strains.

Nucleic acid /Amino acid	S95-E4 Parent *	S95 Vaccine	B3389	B3273	B3510	B3639	B3362	B3616	B3691	B3539	B3364	B3024
ORF1ab (R)	100/100	99/99	99/99	97/97	91/95	97/95	99/99	92/95	99/99	97/98	90/94	95/95
ORF2 (S)	100/100	99/99	99/99	99/99	99/99	99/99	99/99	99/99	99/99	99/99	99/99	97/98
ORF3a	100/100	100/100	100/100	100/100	100/100	92/91	100/100	100/100	100/100	87/85	97/100	91/89
ORF3b	100/100	100/100	100/100	100/100	100/100	100/100	100/100	100/100	100/100	85/78	90/89	92/92
ORF3c (E)	100/100	100/100	100/100	100/100	100/100	100/100	100/100	100/100	100/100	91/93	85/84	85/88
ORF4a (M)	100/100	100/100	100/100	99/100	99/100	99/99	94/97	96/96	97/96	95/97	90/93	90/92
ORF4b	100/100	100/100	100/100	100/100	100/100	100/100	95/93	95/93	93/90	95/93	93/90	86/82
ORF5a	100/100	99/98	99/98	98/96	98/98	99/98	93/92	93/93	93/90	93/93	93/90	84/81
ORF5b	100/100	100/100	100/100	100/100	100/100	100/100	99/100	100/100	96/96	98/97	96/96	90/87
ORF6a (N)	100/100	100/100	100/100	99/99	99/99	100/100	96/98	99/99	96/99	95/96	91/94	94/95
ORF6b	100/100	100/100	100/100	99/98	99/98	100/100	99/98	99/98	73/62	99/98	96/94	99/98
**Nucleic acid** **/Amino acid**	**S95-E4 Parent**	**S95** **Vaccine** ^#^	**B3389**	**B3273**	**B3510**	**B3639**	**B3362**	**B3616**	**B3691**	**B3539**	**B3364**	**B3024**
ORF1ab (R)	99/99	100/100	99/99	97/97	94/95	97/95	99/99	94/95	98/99	94/98	93/94	95/95
ORF2 (S)	99/99	100/100	99/99	99/99	99/99	98/99	99/99	99/99	99/99	99/99	98/99	97/98
ORF3a	100/100	100/100	100/100	100/100	100/100	92/91	100/100	100/100	100/100	87/85	97/100	91/89
ORF3b	100/100	100/100	100/100	100/100	100/100	100/100	100/100	100/100	100/100	85/78	90/89	92/92
ORF3c (E)	100/100	100/100	100/100	100/100	100/100	100/100	100/100	100/100	100/100	91/93	85/84	85/88
ORF4a (M)	100/100	100/100	100/100	99/100	99/100	99/99	94/97	96/96	97/96	95/97	90/93	90/92
ORF4b	100/100	100/100	100/100	100/100	100/100	100/100	95/93	95/93	93/90	95/93	93/90	86/82
ORF5a	99/98	100/100	100/100	99/98	99/98	100/100	92/90	92/92	92/89	93/92	92/89	84/81
ORF5b	100/100	100/100	100/100	100/100	100/100	100/100	99/100	100/100	96/96	98/97	96/96	90/87
ORF6a (N)	100/100	100/100	100/100	99/99	99/99	100/100	96/98	99/99	96/98	95/96	91/94	94/95
ORF6b	100/100	100/100	100/100	99/98	99/98	100/100	99/98	99/98	73/62	99/98	96/94	99/98

Identity (%) of the eleven IBV genes compared to those of the S95 parent strain (top) or S95 vaccine strain (bottom) is shown, separated by a slash, as nucleotide sequence identity/amino acid identity. Columns exhibiting 100% identity in both nucleotide and amino acid sequences are highlighted in blue, while those with 100% identity solely in amino acid sequences are distinguished in purple. * Indicates the S95 parent strain as 100%. ^#^ indicates the S95 vaccine strain as 100%.

**Table 3:** Amino acids that differ between the S95-E4 parent strain and the S95 vaccine strain, and their amino acids in the S95 vaccine-like IBV strains.

Gene	Position	S95-E4 Parent	S95 Vaccine	B3389	B3273	B3510	B3639	B3362	B3616	B3691
ORF1ab (R) 5/6633	3928	Ser	Phe	Phe	Ser	Ser	Phe	Ser	Ser	Phe
	3939	Asp	Tyr	Tyr	Asp	Glu	Asp	Asp	Gly	Gly
	4346	His	Gln	Gln	His	His	His	His	His	His
	4851	Ser	Asn	Asn	Ser	Ser	Ser	Ser	Ser	Ser
	5382	Thr	Ile	Ile	Thr	Thr	Thr	Thr	Thr	Thr
ORF2 (S) 3/1169	123	Pro	Leu	Pro	Pro	Pro	Pro	Pro	Pro	Pro
	370	Phe	Leu	Phe	Phe	Phe	Phe	Phe	Phe	Phe
	1161	Glu	Stop	Glu	Stop	Glu	Glu	Glu	Glu	Glu
ORF5a (NS) 1/65	11	Val	Ala	Ala	Ala	Val	Ala	Val	Val	Val

The number shown for each gene indicates the number of different amino acids between the S95-E4 parent and the S95 vaccine strains per total amino acids of the encoded protein. Amino acids at the indicated positions of *ORF 1ab (R)*, *ORF2 (S)*, and *ORF5a (NS)* genes are shown by a three-letter code. Amino acids that are the same as the S95-E4 parent strain’s are indicated in blue, while the amino acids that are different are indicated in orange or white.

## Data Availability

The datasets analyzed during the current study are available from the corresponding author upon reasonable request.

## Supplementary Materials

The following supporting information can be downloaded at: https://www.mdpi.com/article/10.3390/vaccines13111092/s1, Figure S1: Egg embryo mortality associated with the B3389 strain (retest); Figure S2: Replication capacity of the B3389 strain in embryonated chicken egg (retest).
