# Supplementary figures and images for: Sporadic Outbreaks of Avian Infectious Bronchitis Viruses Highly Similar to the S95 Live Attenuated Vaccine Strain in Japan: A Comparative Study of Ten Field Isolates and S95
Source: Vaccines (Basel). 2025 Oct 24;13(11):1092. doi: 10.3390/vaccines13111092 (PMC12656252; doi:10.3390/vaccines13111092)

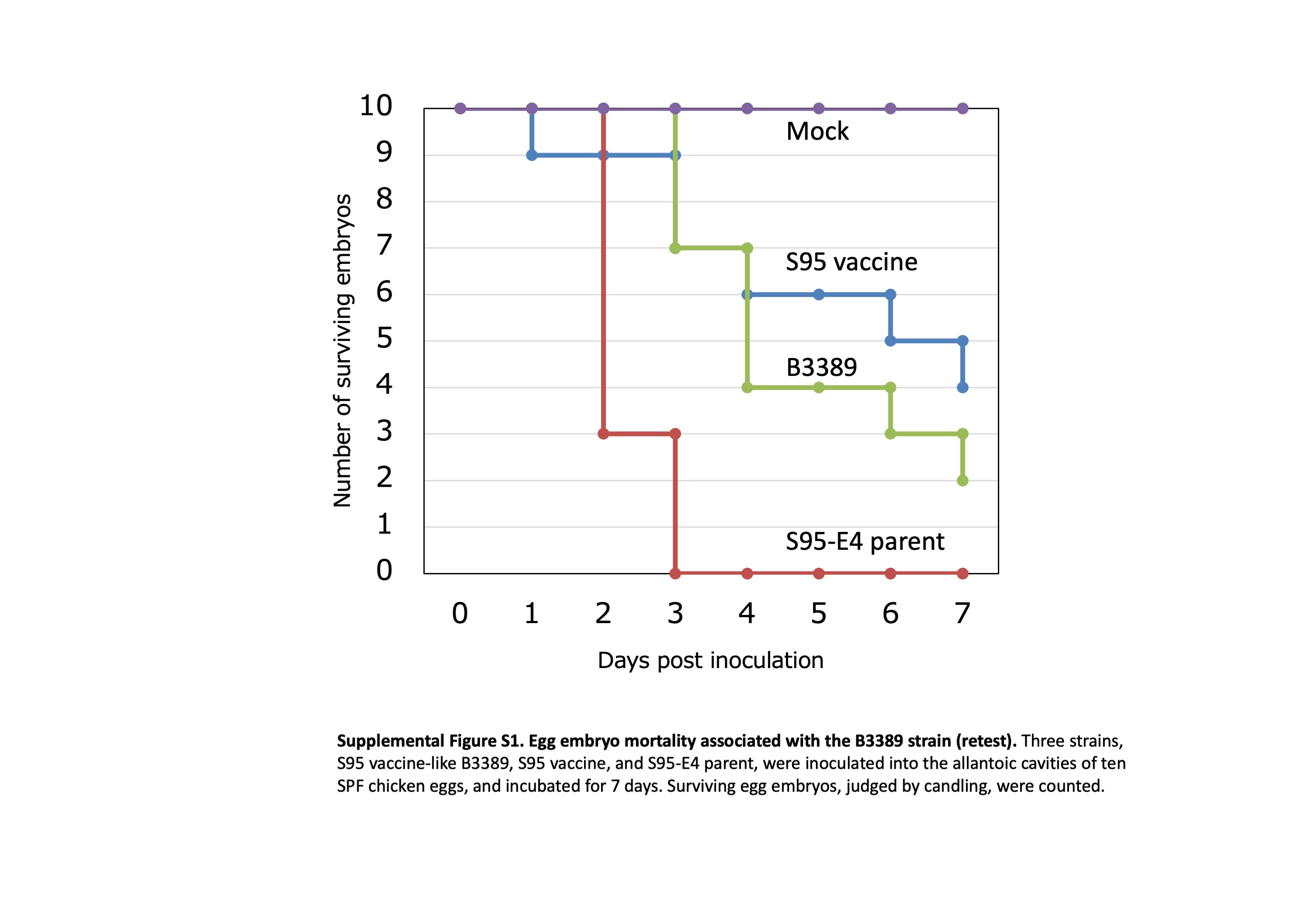

Supplement: Supplementary file 1 [file vaccines-13-01092-s001.zip › Supplementary Figures/Supplemetary Figure S1.png]

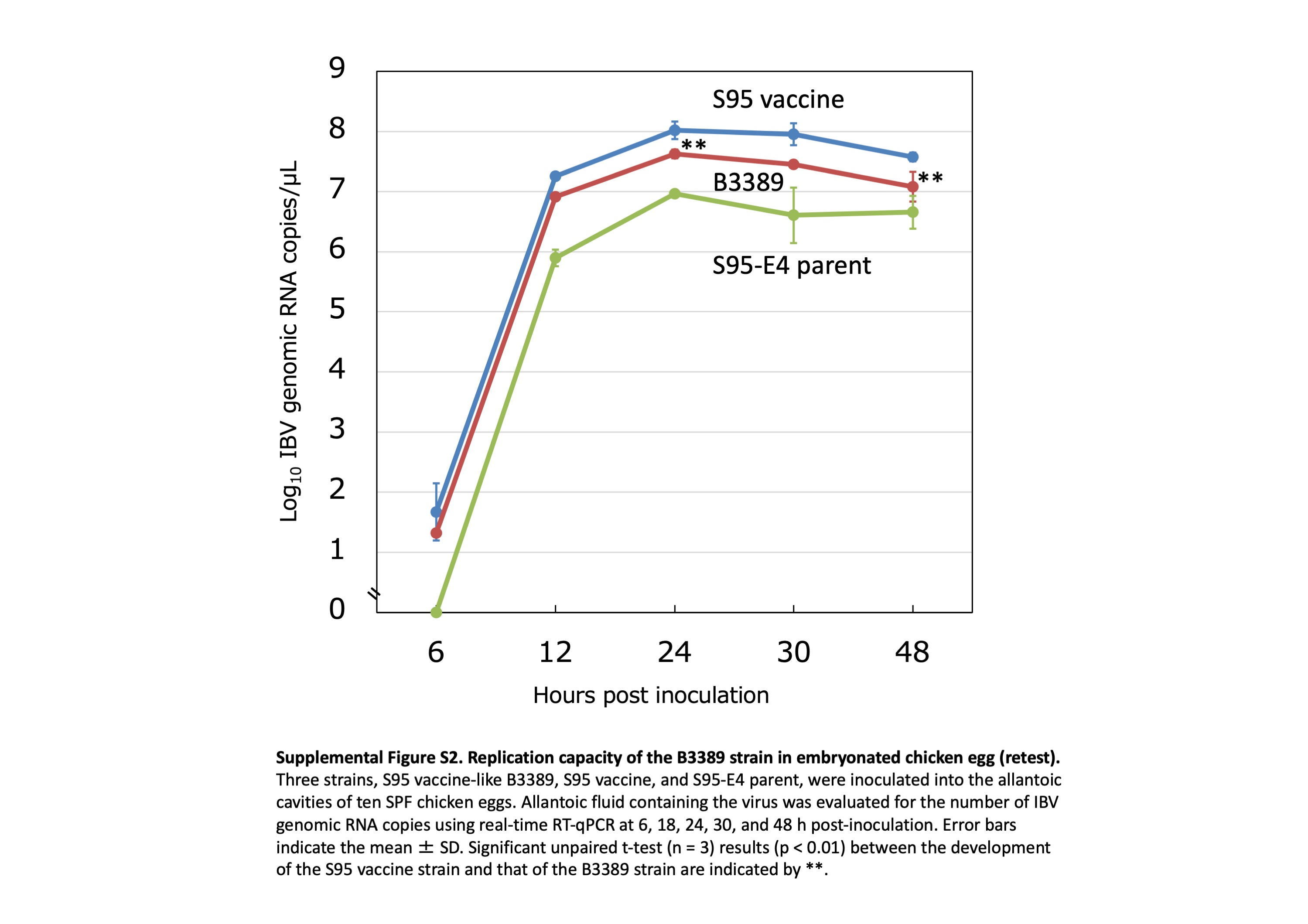

Supplement: Supplementary file 1 [file vaccines-13-01092-s001.zip › Supplementary Figures/Supplemetary Figure S2.png]
